# Effect of Physical Therapy on Vestibular Function in Body Lateropulsion Following Wallenberg Syndrome: A Case Report Using the Modified Clinical Test of Sensory Interaction and Balance

**DOI:** 10.7759/cureus.98212

**Published:** 2025-12-01

**Authors:** Eisei Harayama, Yuri Hayashi, Shota Tanaka, Kota Yamauchi, Shuji Arakawa

**Affiliations:** 1 Department of Behavior and Health Sciences, Kyushu University, Fukuoka, JPN; 2 Department of Rehabilitation, Steel Memorial Yawata Hospital, Kitakyushu, JPN; 3 Department of Rehabilitation Medicine, Kyushu University Hospital, Fukuoka, JPN; 4 Stroke and Neurological Center, Steel Memorial Yawata Hospital, Kitakyushu, JPN

**Keywords:** body lateropulsion, case report, modified clinical test of sensory interaction and balance, vestibular function, wallenberg syndrome

## Abstract

Balance training, consisting of postural control exercises, has been shown to enhance vestibular-related postural function in individuals with body lateropulsion (BL), a condition characterized by abnormal lateral displacement of body mass and increased lateral sway when estimating the center of pressure at rest. We report a single case showing quantitative and longitudinal changes in estimated vestibular-related postural function using the modified Clinical Test of Sensory Interaction and Balance (mCTSIB), which evaluates postural control by isolating vestibular input while controlling for visual and somatosensory influences. A 52-year-old male presented with right-sided BL and was diagnosed with lateral medullary infarction (Wallenberg syndrome). Vestibular-related postural function was assessed repeatedly using the mCTSIB during a structured physical therapy program that included postural control and balance training. Initially, the patient exhibited right-sided BL and ataxia, requiring assistance with ambulation due to a deviation in subjective visual vertical (SVV). Over time, SVV normalized, corresponding with improvements in the estimated vestibular function area derived from the maximum entropy method within the 0.2-2.0 Hz range on the mCTSIB. The patient ultimately regained independence in activities of daily living, and no adverse events occurred during therapy. This case suggests that postural control training may be associated with the recovery of vestibular-related postural function in individuals with BL. Quantitative assessment using the mCTSIB may provide a valuable framework for monitoring vestibular rehabilitation outcomes. However, as this observation is based on a single case, the findings should be interpreted cautiously and warrant further validation through larger studies.

## Introduction

Wallenberg syndrome, caused by dorsolateral medullary infarction, typically presents with dizziness, ataxia, and dissociated sensory loss affecting the face ipsilateral to the lesion and the trunk and limbs contralaterally [1,2]. Among these symptoms, body lateropulsion (BL), an involuntary ipsilateral lean or fall due to vestibular tone imbalance, is a key and clinically relevant feature [1-3].

BL reflects an impaired ability to maintain and control upright posture and may accompany an ocular tilt reaction (OTR), a pathological eye tilt toward the lesion side [1,4]. Although BL can result from damage to brainstem structures such as the pons or midbrain, or to the cerebellum, it typically occurs ipsilateral to the lesion in dorsolateral medullary infarction. Moreover, BL usually follows a transient course, though delayed presentations have been reported [2]. While the pathophysiological mechanisms of BL are increasingly understood, the characteristics of postural control in affected individuals remain incompletely elucidated. Because BL often impairs standing balance and gait stability, it represents a major challenge during early rehabilitation after brainstem stroke.

The center of pressure (COP) at rest, a measure that quantifies body sway by tracking the point of application of the ground reaction force, is a key parameter in postural control assessment and provides insight into disease-related postural abnormalities. Patients with BL show an abnormal lateral shift or increased lateral sway of the COP, with more pronounced deviations in severe cases [1]. An increased COP velocity toward the affected side is also a distinctive feature, reflecting a tendency to fall toward the lesion side during standing [5,6]. These postural disturbances correlate with BL severity and subjective visual vertical (SVV) tilt, a measure of perceived vertical orientation used to detect vestibular dysfunction [1,2]. Abnormal SVV values indicate impaired vertical perception and serve as a clinical marker of balance dysfunction and vertigo [2,7]. Together, these findings suggest that BL is closely associated with vestibular dysfunction.

The modified Clinical Test of Sensory Interaction and Balance (mCTSIB), a test that assesses standing stability under various sensory conditions, is designed to evaluate vestibular contributions to postural control by manipulating visual and somatosensory inputs. This test assesses standing stability under four sensory conditions [6,8]. Visual input is altered by eye closure, and somatosensory feedback is modified using a foam pad placed under the feet. Individuals with vestibular dysfunction exhibit greater body sway when standing on a compliant surface with eyes closed [8]. Thus, the mCTSIB enables quantitative estimation of vestibular postural control by systematically changing sensory input.

Previous studies have used clinical and semi-quantitative scales, such as the Grading Lateropulsion Scale (a semi-quantitative scale) and the Berg Balance Scale (a functional/clinical scale), to assess BL severity [5,6]. Quantitative analyses have also examined COP deviations to evaluate vestibular function in BL. However, no studies have reported longitudinal quantitative assessment of vestibular function using the mCTSIB, which detects the frequency and magnitude of postural sway and performs power spectrum analysis (Hz) to identify increased power at specific frequencies within the 0.02-10 Hz range [9].

Establishing a quantitative framework for evaluating postural control changes related to vestibular recovery in BL could help optimize rehabilitation strategies and develop targeted physical therapy interventions. This case report aimed to describe the feasibility of quantitative and longitudinal changes in vestibular function using the mCTSIB in a patient with BL. This case report was prepared in accordance with the CARE guidelines.

## Case presentation

**Figure 1:**
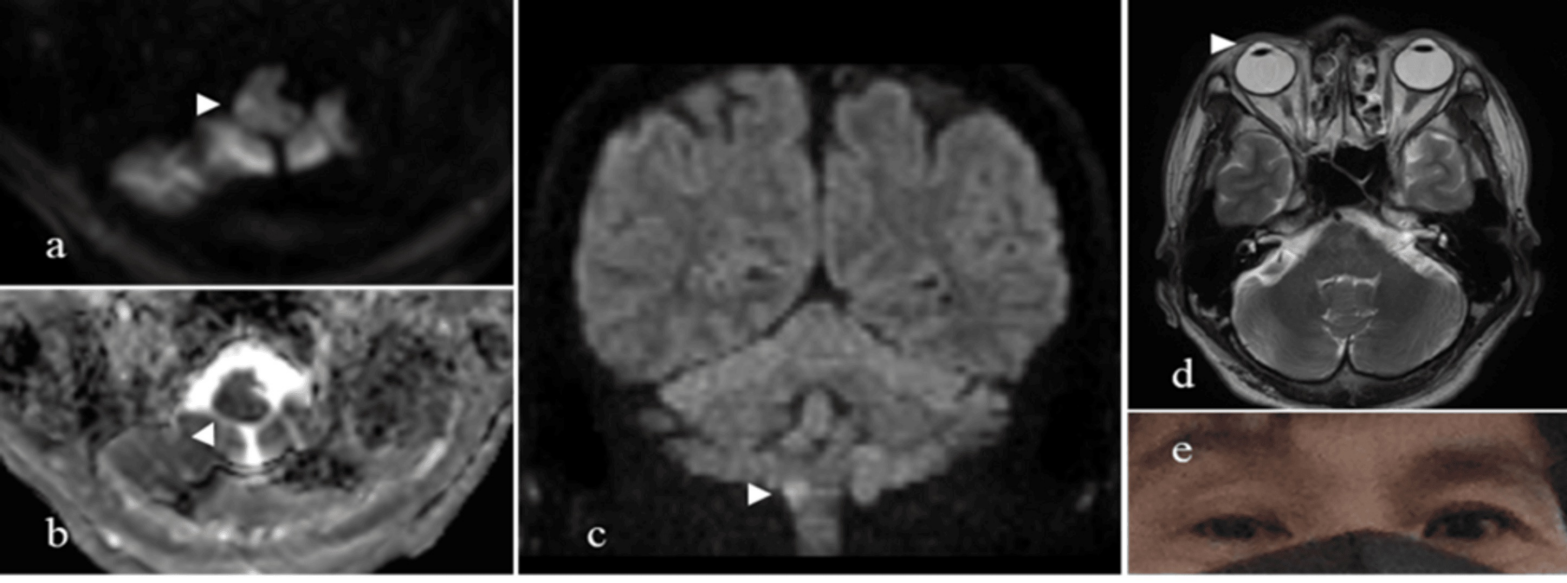
Magnetic resonance imaging findings of the patient (a) Diffusion-weighted imaging (DWI) showing high signal intensity in the right dorsolateral medulla indicating an acute infarction. (b) Apparent diffusion coefficient (ADC) map demonstrating corresponding low signal intensity. (c) Additional DWI confirming the area of infarction. (d) Ocular tilt reaction (OTR) observed in the right eye. (e) Deviation of eye position indicating vestibular imbalance. Arrowheads indicate the site of infarction and gaze deviation.

A 52-year-old male patient presented to the emergency room with a sensation of floating dizziness and a headache in the right temporal region while at work, along with numbness and mild weakness in the right upper and lower limbs, nausea, and difficulty walking. The patient had no underlying medical conditions, nor did he have a history of alcohol consumption or smoking. Upon arrival, he was alert and oriented and exhibited sensory deficits on the right side of his face and the left side of his trunk, upper limb, and lower limb. Consequently, his National Institutes of Health Stroke Scale (NIHSS) score was 1. Magnetic resonance imaging revealed high signal intensity on diffusion-weighted imaging (DWI) and low signal intensity on apparent diffusion coefficient (ADC) mapping, as well as an OTR in the right medulla oblongata (Figure 1). Magnetic resonance angiography showed a dissection of the right vertebral artery. The patient was diagnosed with Wallenberg syndrome secondary to vertebral artery dissection.

Physical therapy intervention

Physical therapy began two days after symptom onset and was delivered by a physical therapist with more than five years of experience in vestibular rehabilitation. The neurologist recommended physical therapy with gradual mobilization; however, the patient was unable to mobilize until the fifth day due to dizziness and nausea. On the sixth day, the patient was able to mobilize, and physical therapy was initiated. At that time, he reported experiencing involuntary lateral displacement of his body toward the right side and exhibited right-sided BL. On stance assessment, he leaned >10° to the right; no Horner’s syndrome or nystagmus was observed. This tendency to fall to the right side was exacerbated when his eyes were closed.

Physical therapy for BL was conducted for 60 minutes per day, five times per week. The intervention included standard rehabilitation exercises, such as balance training, muscle-strengthening exercises, gait training, and activities of daily living (ADL) exercises. Balance training included progressive postural control exercises with visual feedback. The exercises included static standing, single-leg standing on a balance mat, eyes-closed standing, and tandem walking. The patient progressed to the next exercise (e.g., from static standing to single-leg standing on a balance mat or eyes-closed standing, and then to tandem walking) once he was able to maintain balance independently without physical assistance and could perform the exercise stably across multiple trials.

Clinical scales and outcome measures

According to the severity scale proposed by Dieterich and Brandt, the patient was classified as grade IV (falling with eyes open, head and trunk tilted). The Burke Lateropulsion Scale (BLS), which evaluates resistance during transitions from a supine position to walking, was used. The Stroke Impairment Assessment Set (SIAS) [10] was administered to evaluate global function after stroke (maximum score: 76, higher scores indicate better function). Although cognitive function was intact, the patient exhibited ataxia, which was evaluated using the Scale for the Assessment and Rating of Ataxia (SARA). SARA is a 40-point scale with eight items: walking, standing, sitting, speech, finger-chase, finger-nose, hand pronation/supination, and heel-toe coordination. Walking independence was assessed using the Functional Ambulation Categories (FAC) [11], and ADL performance was evaluated using the Barthel Index (BI).

Measurement of standing postural control

To assess vestibular-related contributions to postural control, the mCTSIB was conducted using a stabilizer (Gravicorder GW-31; Anima Co., Ltd., Tokyo, Japan). Power spectrum analysis was performed, with the primary output measured in hertz (Hz). Postural stability was evaluated under four conditions: (i) condition 1, with eyes open on a solid surface, (ii) condition 2, with eyes closed on a solid surface, (iii) condition 3, with eyes open on a foam pad, and (iv) condition 4, with eyes closed on a foam pad.

In each condition, the patient's feet were positioned with toes 30° apart, heels together, and arms resting at the sides for 60 seconds. Each mCTSIB condition was performed once for 60 seconds; COP and maximum entropy method (MEM) values were derived from a single trial due to patient fatigue. A foam pad was placed on the force platform to provide a compliant surface, and the COP was calculated from the ground reaction forces and moments in the anterior-posterior and medial-lateral directions and analyzed in the time-frequency domain [9].

Each frequency band (Hz) was associated with musculoskeletal, cerebellar, vestibular-related, or visual contributions to postural control. The local energy content of each frequency band was calculated, and the MEM for left-right and anterior-posterior sway was expressed as a percentage under both eyes-open and eyes-closed conditions. Left-right MEM was specifically analyzed due to its relevance to lateral body lateropulsion and vestibular-related postural control. MEM is a technique used to determine the spectrum with the highest information entropy relative to an unknown autocorrelation function, serving as an index of information ambiguity.

Subjective visual vertical measurement

**Figure 2:**
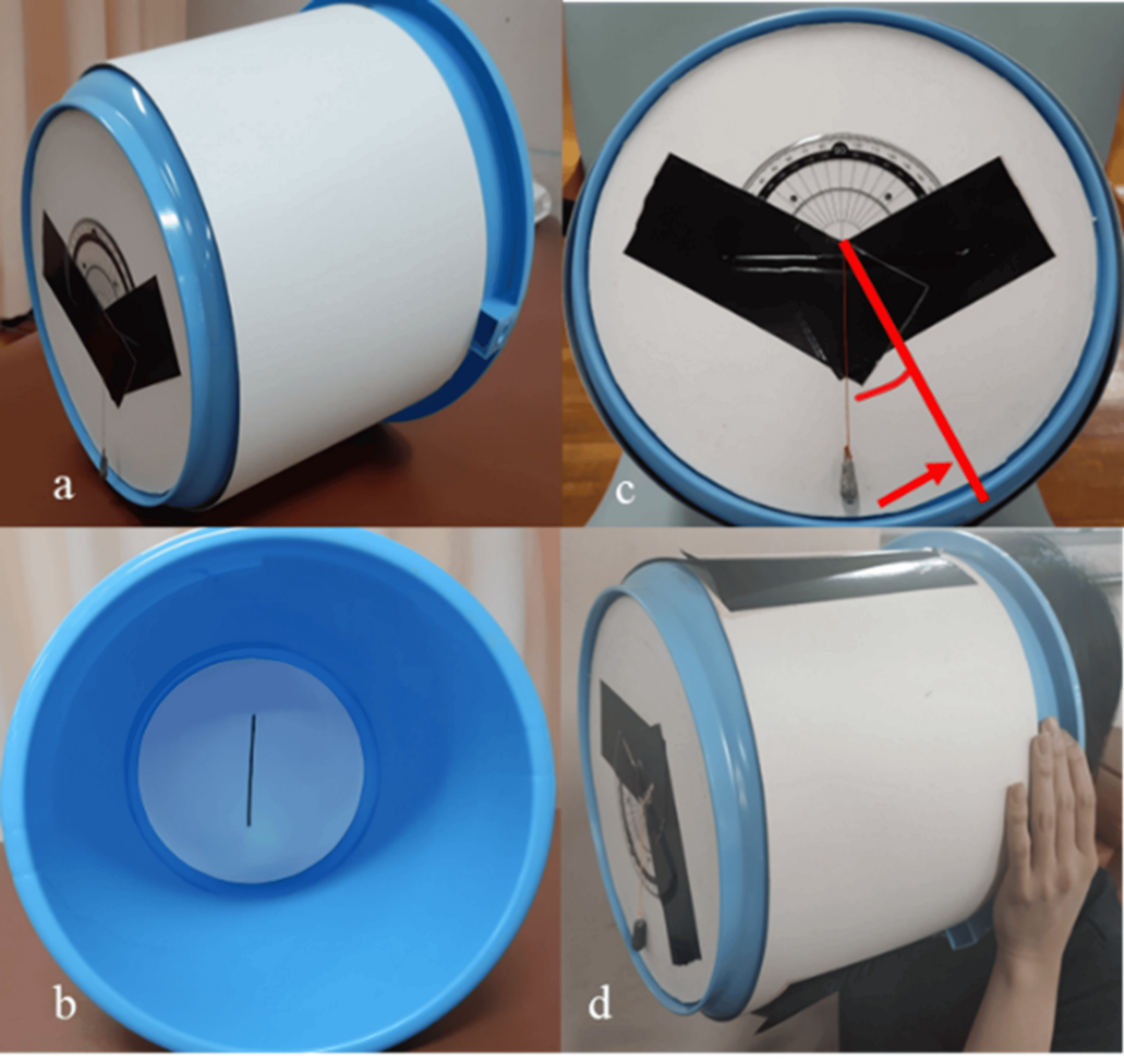
Assessment of subjective visual vertical (SVV) using the bucket method A bucket equipped with an internal protractor is used to measure the tilt angle of perceived verticality by the patient

The bucket method [7,12] was used to assess SVV. The patient looked into a bucket equipped with a grading device and was instructed to align a straight line visible on the inner bottom of the bucket to what he perceived as vertical (Figure 2). The examiner rotated the bucket’s inner bottom randomly, and an angle protractor attached to the outer bottom allowed for precise measurement of the tilt angle. The patient completed 10 trials, and the average deviation was calculated.

Results

The patient completed the physical therapy program without any adverse events. Serial measurements were obtained at T1 (12 days after onset), T2 (20 days), and T3 (36 days, at discharge) (Table 1). At T1, clinical scale scores were as follows: BLS, 6; SIAS, 67; SARA, 12.5; FAC, 2; and BI, 75. The BLS indicated marked resistance during standing, transfers, and walking. The SARA revealed positive findings on the finger-to-nose and heel-to-shin tests, with ataxia observed during standing and ambulation. Consequently, the patient required assistance with ADL. At T2, scores improved to BLS, 0; SIAS, 76; and SARA, 2. Both ataxia severity and movement resistance had improved. Consequently, the FAC score increased to 4, indicating greater walking independence, and the BI showed further recovery. By T3, the SARA score had normalized (0), ataxic symptoms had resolved, and the BI score reached 100, indicating full independence in ADL.

**Table 1:** Changes in the clinical scales and measures T1: Day 12; T2: Day 20; T3: Day 36; NM: Not Measured, BLS: Burke Lateropulsion Scale; SIAS: Stroke Impairment Assessment Set; SARA: Scale for the Assessment and Rating of Ataxia; FAC: Functional Ambulation Categories; BI: Barthel Index; SVV: Subjective Visual Vertical.

Variable	Day 6	T1	T2	T3
BLS (points)	NM	6	0	0
SIAS (points)	67	67	76	76
SARA (points)	12.5	12.5	2	0
FAC (category)	2	2	4	5
BI (points)	50	75	85	100
SVV (°)	NM	4 ± 2	2 ± 1	1 ± 1

Standing postural control measurements

**Figure 3:**
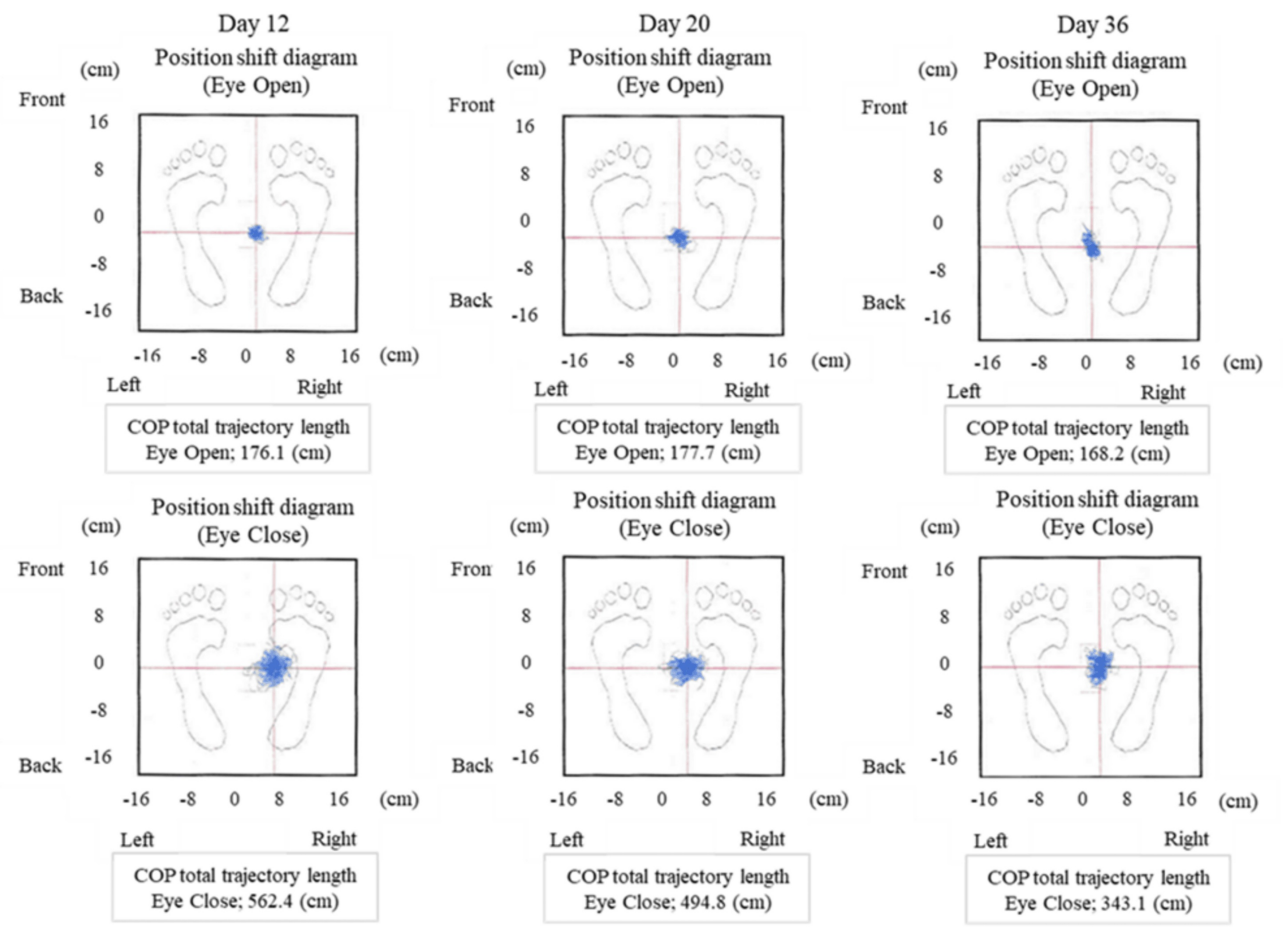
Changes in the total trajectory length of the center of pressure (COP) over time Trajectory length of the COP was measured at three time points during rehabilitation to evaluate postural control.

Postural control assessments demonstrated improvement in the total trajectory length of the COP (cm) from T1 to T3 (Figure 3).

**Figure 4:**
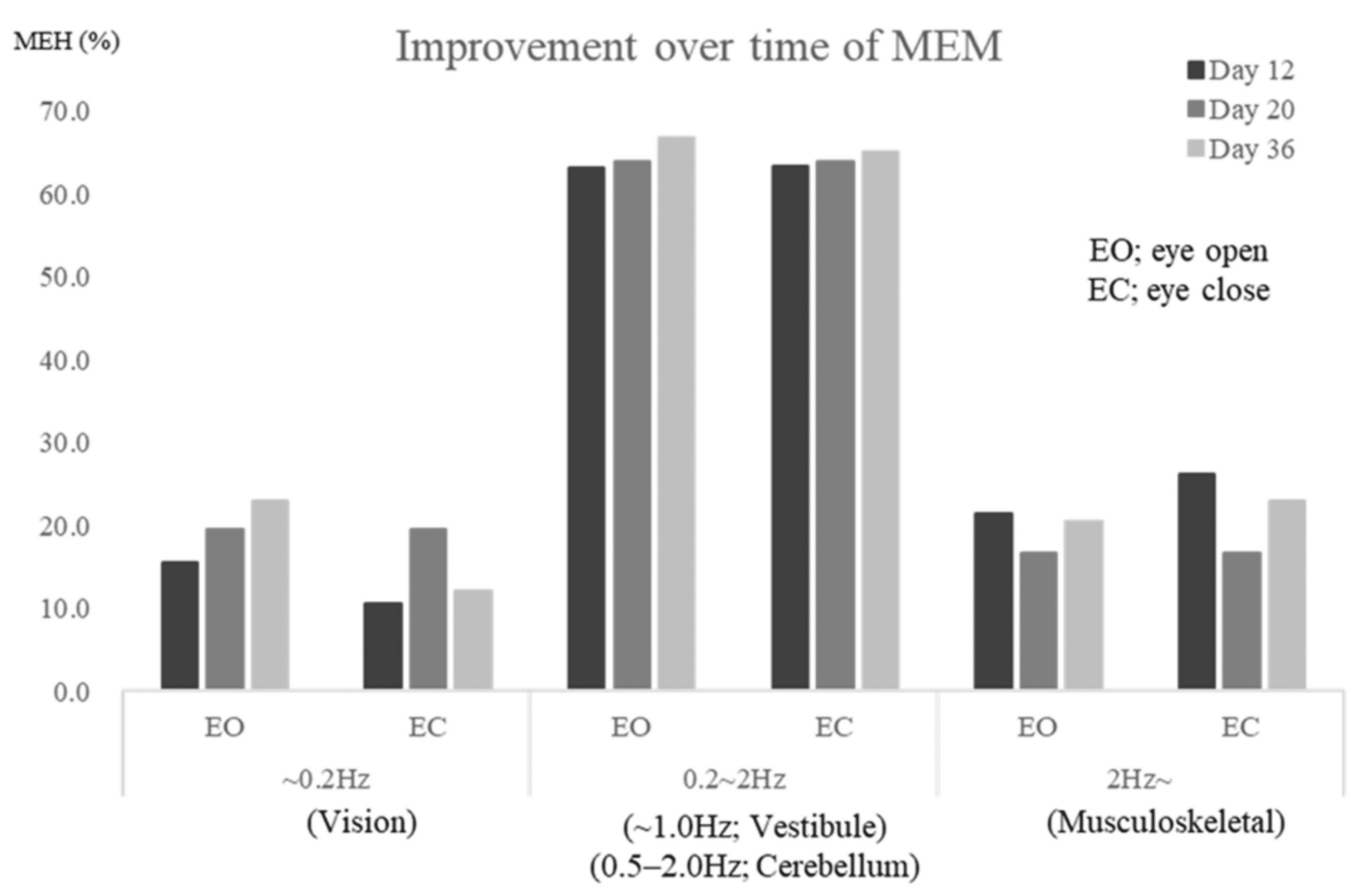
Time-frequency analysis using the maximum entropy method (MEM) Spectral analysis of postural sway was conducted using the MEM to detect vestibular-related frequency changes.

Power spectrum analysis of the COP showed an increase in MEM within the vestibular-related contribution to postural control range (0.2-2.0 Hz) from T1 to T3 under the eyes-open condition (Figure 4). Vestibular-band MEM (0.2-2.0 Hz, eyes open) increased from 18% (T1) to 42% (T3), a 133% relative gain. There was also a trend toward increased MEM at 0.2 Hz and decreased MEM at 2.0 Hz over time. SVV, assessed using the bucket method, was initially deviated 4 ± 2° toward the right (ipsilesional) side at T1, improving to 1 ± 1° from T2 onward (Table 1). Each mCTSIB condition was performed once for 60 s; COP and MEM values were derived from a single trial due to patient fatigue.

## Discussion

The patient in this case report developed right-sided BL due to a lateral medullary infarction. We evaluated changes in vestibular-related contributions to postural control using the mCTSIB in a patient with BL following physical therapy. Initially, the patient exhibited a rightward falling tendency when standing. The semi-quantitative BLS score was 6, indicating lateropulsive resistance during standing, transfers, and walking. The SARA score was 12.5, and limb ataxia was present. Additionally, the SVV was tilted 4° toward the lesion side, suggesting an altered perception of verticality. The estimated MEM and COP measurements at baseline during mCTSIB testing indicated reduced vestibular-related contributions to postural control.

The physical therapy intervention followed a standardized program, including balance exercises incorporating postural control training with visual feedback. Following the intervention, MEM values within the vestibular-related contribution to postural control range (0.2-2.0 Hz) progressively increased, suggesting improvements in postural control supported by vestibular inputs (Figure 4). Additionally, MEM at 0.2 Hz increased under the eyes-open condition, while MEM at 2 Hz decreased, suggesting increased visual compensation and reduced reliance on the musculoskeletal system.

Postural control is maintained through integrated sensory inputs from the visual, vestibular, cerebellar, and proprioceptive systems. When one sensory modality is impaired, the remaining systems compensate by increasing their contribution to postural control [13]. Previous studies have reported that BL severity is heightened in vestibular-dominant standing positions, particularly when visual and somatosensory inputs are attenuated [14]. Similar to prior reports, this patient exhibited BL toward the lesion side due to right lateral medullary infarction. mCTSIB testing showed an elongated total trajectory length of the COP under eyes-closed conditions, with reduced MEM values within the 0.2-2.0 Hz range at baseline.

**Figure 5:**
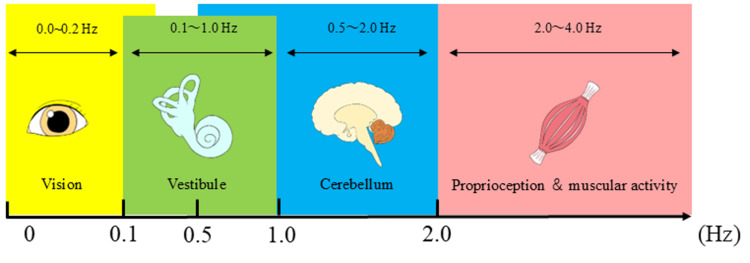
Conceptual model of sensory system contributions to postural control based on sway frequency. Illustrates the generally accepted conceptual model associating specific postural sway frequency ranges with the dominant sensory systems (vision, vestibular, cerebellum, and proprioception/muscular activity). Image Credit: Authors; based on the concepts and findings reported in Zijlstra et al. [9], Baloh et al. [15], Diener et al. [16], and Yokoyama et al. [17]

Power spectrum analysis has previously shown that lateralization of frequencies below 1 Hz suggests a vestibular input disorder [15], enhanced sway at approximately 0.2 Hz indicates proprioceptive or visual dysfunction [16], and increased sway within the 2-4 Hz range is associated with cerebellar dysfunction (Figure 5) [17]. In this case, spectral analysis findings suggested a vestibular-related postural control disorder at baseline. However, as vestibular-related contributions to postural control improved over time, reliance on the musculoskeletal system (≥2 Hz) decreased.

MEM within the visual range (<0.2 Hz) was initially low but increased over time, suggesting an influence of the OTR. In addition to BL-related postural control impairments, the patient exhibited ataxia and an SVV tilt, indicative of altered vertical perception. Individuals with BL and limb ataxia have been reported to exhibit impaired proprioceptive input due to disruption of the ascending dorsal vestibulo-spino-cerebellar tract [18]. At baseline, the SVV tilt was 4° toward the lesion side. The bucket test for SVV assessment has been validated against the hemispheric dome test, with inter- and intra-examiner reliability of 0.9 [7,12]. The normal SVV range is -2.58° to 2.58° [2,7,12]. The SVV tilt is considered a highly sensitive indicator of vestibular tone imbalance [4]. This patient exhibited vertical perception abnormalities at baseline, as evidenced by the SARA and SVV assessments. In patients with stroke and brainstem lesions, SVV tilting toward the affected side is believed to result from asymmetric tone within the torsional vestibulo-ocular pathway [3]. However, as rehabilitation progressed, ataxia improved, and vertical misperception normalized early in the recovery process (Table 1). This trajectory is consistent with SVV normalization patterns reported by Dieterich and Brandt in patients with BL [1,2], suggesting that this case was not one of delayed BL. Rather, the patient demonstrated early BL improvement, which may have influenced postural control responses during mCTSIB assessments.

The physical therapy program primarily included postural control training with visual feedback. Rehabilitation interventions, particularly visual balance training targeting vestibular function, resulted in MEM improvements within the sub-0.2 Hz range under the eyes-open condition, as well as a reduction in the total COP trajectory length (Figure 3). Each mCTSIB condition was performed once due to patient fatigue, which limited the resolution for isolated analysis of the 1 Hz frequency band specifically associated with vestibular input. These findings suggest that balance mat exercises contributed to improvements in vestibular-related contributions to postural control. Exercise therapy on balance mats has been shown to enhance activity within the vestibular cortex [19], and it is possible that this mechanism facilitated vestibular function improvements in this patient. Following the intervention, reliance on visual and musculoskeletal compensatory mechanisms was decreased, and MEM indices reflecting vestibular-related postural control improved, indicating a more stable and efficient integration of vestibular input for postural control.

While semi-quantitative assessments such as the BLS and SARA are useful for evaluating functional limitations and ADL performance, the mCTSIB objectively estimates vestibular-related contributions to postural control. This case highlights the potential of mCTSIB as a valuable tool for quantifying vestibular function improvements and assessing the effectiveness of vestibular rehabilitation strategies.

Limitations

In this case, the efficacy of the physical therapy intervention for BL was assessed by examining changes in Hz over time using time-frequency domain analysis with the mCTSIB. However, power spectrum analysis was limited to the 0.2-2.0 Hz range, precluding isolated analysis of 1 Hz activity within the vestibular-related contribution to postural control. This limitation was due to the resolution constraints of the measurement equipment, which did not allow for separate analysis of a single frequency component. As a result, the findings did not provide a definitive demonstration of improvements in vestibular-related contributions to postural control. Therefore, these results should be interpreted with caution.

Additionally, this was a single-case study, and statistical significance differences could not be determined. Consequently, it remains unclear whether the changes in Hz values in the mCTSIB from T1 to T3 represent a significant improvement. Furthermore, the equipment used to perform mCTSIB measurements in this case differed from that used in previous studies [4,5,14]. As a result, direct comparisons with prior research may be limited.

## Conclusions

Physical therapy interventions focused on balance training were associated with improvements in vestibular-related contributions to postural control in a patient with BL due to lateral medullary infarction. The frequency-specific analysis using the mCTSIB shows promise as a tool for quantifying individual sensory system contributions to postural control. Specifically, this case suggests that time-frequency analysis focusing on variations within the vestibular-related contribution to postural control frequency range (0.2-2.0 Hz) using the mCTSIB may provide an objective method for tracking functional recovery in BL. This approach offers insights beyond traditional postural indices and could support the development of targeted, frequency-specific physical therapy programs for patients with lateral medullary infarction. Further studies with larger cohorts are warranted to confirm these findings.
